# Pre-, Per- and Postoperative Factors Affecting Performance of Postlinguistically Deaf Adults Using Cochlear Implants: A New Conceptual Model over Time

**DOI:** 10.1371/journal.pone.0048739

**Published:** 2012-11-09

**Authors:** Diane S. Lazard, Christophe Vincent, Frédéric Venail, Paul Van de Heyning, Eric Truy, Olivier Sterkers, Piotr H. Skarzynski, Henryk Skarzynski, Karen Schauwers, Stephen O'Leary, Deborah Mawman, Bert Maat, Andrea Kleine-Punte, Alexander M. Huber, Kevin Green, Paul J. Govaerts, Bernard Fraysse, Richard Dowell, Norbert Dillier, Elaine Burke, Andy Beynon, François Bergeron, Deniz Başkent, Françoise Artières, Peter J. Blamey

**Affiliations:** 1 Bionics Institute, Melbourne, Australia; 2 Service d'otologie et d'otoneurologie, Hôpital R.-Salengro, CHRU de Lille, Lille, France; 3 CHU Gui de Chauliac, Service d'ORL et Chirurgie Cervico-Faciale, Montpellier, France; 4 Department of Otorhinolaryngology and Head and Neck Surgery, Antwerp University Hospital, University of Antwerp, Antwerp, Belgium; 5 Hospices Civils de Lyon, Hôpital Edouard Herriot, Département d'ORL, de Chirurgie Cervico-Maxillo-Faciale et d'Audiophonologie, Lyon, France; 6 AP-HP, Hôpital Beaujon, Service d'ORL et Chirurgie Cervico-Faciale, Clichy, France; 7 Institute of Physiology and Pathology of Hearing, Warsaw, Poland; 8 Institute of Sensory Organs, Kajetany, Poland; 9 The Eargroup, Antwerp, Belgium; 10 Department of Otolaryngology, The University of Melbourne Cochlear Implant Clinic, The Royal Victorian Eye and Ear Hospital, Melbourne, Australia; 11 University of Manchester, Central Manchester University Hospitals NHS Foundation Trust, Manchester, United Kingdom; 12 University of Groningen, University Medical Center Groningen, Department of Otorhinolaryngology/Head and Neck Surgery, Cochlear Implant Center Northern Netherlands, Groningen, The Netherlands; 13 Department of Otorhinolaryngology, University Hospital of Zurich, Zurich, Switzerland; 14 Hôpital Universitaire Purpan, Service d'ORL et Chirurgie Cervico-Faciale, Toulouse, France; 15 St Thomas' Hospital, Auditory Implants Department, London, United Kingdom; 16 Otorhinolaryngology, Radboud University Nijmegen Medical Center, Mijmegen, The Netherlands; 17 Faculté de médecine, Université Laval, Québec City, Québec, Canada; 18 Graduate School of Medical Sciences (Research School of Behavioural and Cognitive Neurosciences), University of Groningen, Groningen, The Netherlands; 19 Institut Saint Pierre, Service d'Audiophonologie et ORL, Palavas les flots, France; University of Salamanca-Institute for Neuroscience of Castille and Leon and Medical School, Spain

## Abstract

**Objective:**

To test the influence of multiple factors on cochlear implant (CI) speech performance in quiet and in noise for postlinguistically deaf adults, and to design a model of predicted auditory performance with a CI as a function of the significant factors.

**Study Design:**

Retrospective multi-centre study.

**Methods:**

Data from 2251 patients implanted since 2003 in 15 international centres were collected. Speech scores in quiet and in noise were converted into percentile ranks to remove differences between centres. The influence of 15 pre-, per- and postoperative factors, such as the duration of moderate hearing loss (mHL), the surgical approach (cochleostomy or round window approach), the angle of insertion, the percentage of active electrodes, and the brand of device were tested. The usual factors, duration of profound HL (pHL), age, etiology, duration of CI experience, that are already known to have an influence, were included in the statistical analyses.

**Results:**

The significant factors were: the pure tone average threshold of the better ear, the brand of device, the percentage of active electrodes, the use of hearing aids (HAs) during the period of pHL, and the duration of mHL.

**Conclusions:**

A new model was designed showing a decrease of performance that started during the period of mHL, and became faster during the period of pHL. The use of bilateral HAs slowed down the related central reorganization that is the likely cause of the decreased performance.

## Introduction

In 1996, a three-stage model of auditory performance over time was described for 800 adult patients with severe to profound deafness who benefited from a cochlear implant (CI) [1]. The factors included in the model were (in order of relative importance) duration of severe to profound hearing loss (s/p HL), age at implantation, age at onset of s/p HL, duration of CI experience, and etiology. This study has been replicated with data from 2251 patients implanted in 15 different centres since 2002 (Blamey et al, in press). The new study used the same methods as in the 1996 study (general linear model) and confirmed the relevance of each factor. However, the relative effect of the factors has changed, including a relatively greater effect of duration of CI experience and age at onset of s/p HL, and relatively reduced importance of duration of s/p HL. These changes may have arisen from changes in the management of hearing loss, better and sustained use of hearing aids (HAs), different cochlear implant selection criteria, and improved CI devices. These changes were likely to have resulted in a higher average residual level of auditory processing and less reorganized cognitive functions in patients immediately prior to cochlear implantation, followed by a greater and more rapid post-operative improvement. Although the individual factors (duration of s/p HL, age at implantation, age at onset of s/p HL, duration of CI experience, and etiology) were all highly statistically significant in both studies, the proportion of the variance in the population accounted for by these factors was relatively small (21% in the 1996 study, and 10% in the more recent study, see Blamey et al, in press, for discussion of the variance difference). New factors (described below) are introduced in the present analysis to explain more of the variance and address additional hypotheses.

We speculated that central modifications might begin with the onset of moderate hearing loss (mHL), defined as the time from which subjects experienced a pure tone average (PTA) loss of more than 40 dB HL, and/or the time of the first use of HAs. It has been shown, in a functional magnetic resonance imaging (fMRI) study of a cohort of ten postlinguistically deaf subjects, that specific brain reorganizations associated with phonological processing in the right posterior superior temporal gyrus/supramarginal gyrus were influenced by the duration of s/p HL and/or the duration of mHL [2]. The hypothesis that mHL might also be an important factor in CI outcome required exploration in a larger group of subjects.

The relaxation of patient selection criteria since 1996 has resulted in a greater proportion of CI recipients with residual hearing [3]–[7]. At the same time, HA technology has improved [8]–[10] and a greater proportion of CI patients are using HAs pre- and post-operatively. Bimodal stimulation, combining electric and acoustic stimulation on the contralateral non-implanted ear, improves speech understanding in noise, sound localization, and music perception compared to the CI used alone [11], [12]. HA use was included in the analyses to investigate the effects of these changes in clinical practice. Once deafness has become severe to profound, residual hearing and the use of HAs maintaining peripheral and central pathways might dampen a deleterious cerebral reorganization in favor of visual processing [13], [14]. Thus we hypothesized that the negative effect of duration of s/p HL on CI outcome might be reduced by the use of HA(s).

The influence of several per- and postoperative factors, such as the surgical approach used (cochleostomy or round window approach) [15]–[18], the depth or angle of insertion of the electrode array [19]–[21], the number of active electrodes [21], [22], have already been addressed. However the samples were small leading to controversial results. The large sample size of 2251 patients in the present study offered the opportunity to investigate the influence of these factors on CI speech performance with greater certainty.

**Figure 1:**
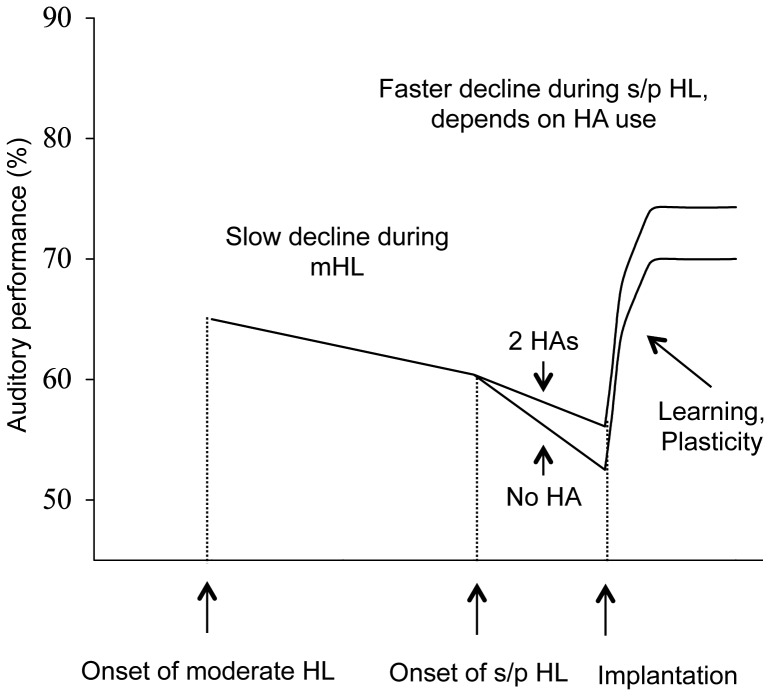
Three-stage model of mean expected auditory performance ranking over time for a hypothetical “average CI recipient”. The detailed description of the Figure is in the Results section. mHL: moderate hearing loss; s/p HL: severe to profound hearing loss, HA: hearind aid.

The aim of the present study was to confirm the new model of auditory performance over time proposed in Figure 1 and to find a sensitive analysis that could test and control the effect of the factors previously outlined, on a large sample of CI recipients (2251). Apart from these factors, gender, level of education, PTA and unaided hearing threshold at 500 Hz of both ears, preoperative speech perception scores, date of surgery, ear chosen for implantation (better or worse ear), and CI brand were also included in the analysis.

## Materials and Methods

Several new statistical analyses were conducted on the dataset described in Blamey et al (in press). The dataset consisted of retrospective information for 2251 CI recipients evaluated with various speech tests and conditions (quiet and noise) from 15 international centres. This project was approved by the Royal Victorian Eye and Ear Hospital Human Research Ethics Committee (Project 10/977H, Multicentre Study Of Cochlear Implant Performance In Adults). In a multicenter study, one of the challenges is to combine information in a useful manner despite the differences in evaluation methods and data recorded in the individual centres. All centres provided the core information on implant performance (on an open-set speech perception test in quiet and in noise without lipreading), duration of s/p HL, age at onset of s/p HL, etiology, and cochlear implant experience. Most centres provided additional information (such as use of HAs before surgery, duration of mHL, and amount of residual hearing) if it was available. The statistical analyses thus included additional factors beyond those used in Blamey et al. (in press) (duration of s/p HL, age at onset of s/p HL, duration of CI experience, and etiology). The number of data points included in each analysis varied because of missing data from some clinics on some factors.

Selection criteria for CI recipients included in the study were: Adult at the time of implantation (>18 years old); Onset of s/p HL after the age of 15 (time from which the patient could no longer use hearing alone to communicate even with the best-fitted hearing aids, and/or understand TV, and/or stopped using the telephone). Four brands of CIs were included (Advanced Bionics, Cochlear, Med-el, and Neurelec). Their proportions in the sample were 21%, 50%, 17%, and 7%, respectively (plus 5% missing data for this variable). Date of implantation was after 2002 for all recipients to include technically comparable improvements across brands.

Speech scores in quiet and in noise at two postoperative times for each recipient were requested from the clinics: one score collected early after activation of the CI (T1) and one score collected later on (T2). The choice of the date of the tests was free and varied between and within centres. The mean and standard deviation for T1 were 0.5 years and 0.8 years, respectively, and 2 years and 1.7 years for T2, respectively.

**Figure 2:**
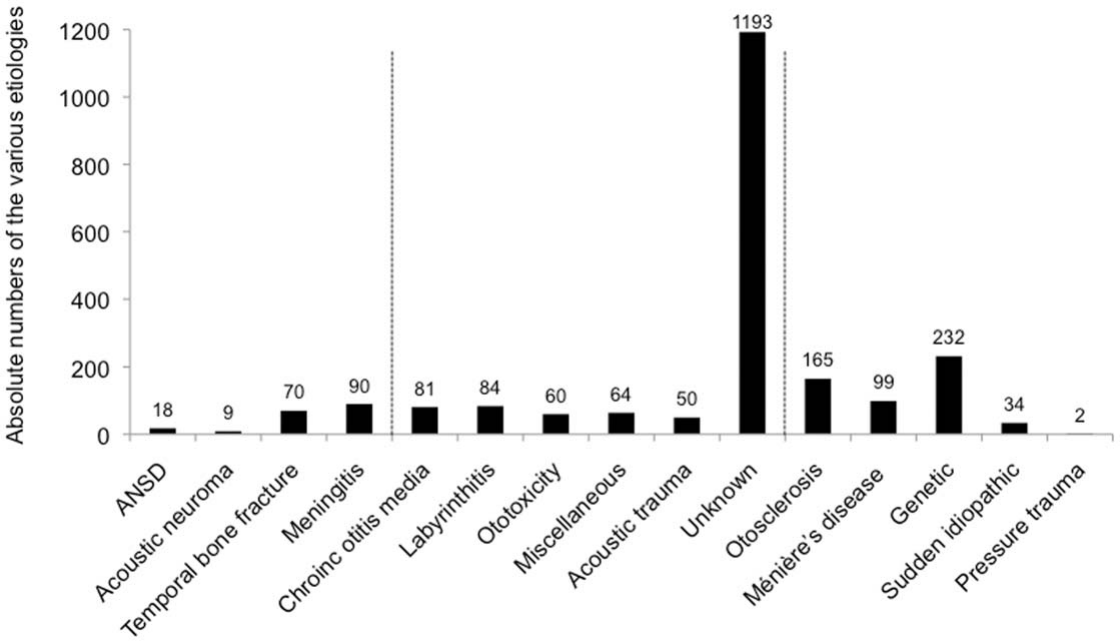
Absolute numbers of the various etiologies defined in the dataset. These etiologies are classified by poorest to best speech outcome in quiet with a CI. ANSD: Auditory neuropathy spectrum disorder. “Miscellaneous” included non-genetic congenital etiologies, cerebral ischemia, drepanocytosis, cephalic trauma without temporal bone fracture, etc. CI recipients presenting with the etiologies encompassed between the two vertical dotted lines showed performances around average, i.e. 50% of speech recognition (not statistically different from average). CI recipients presenting with etiologies on the left part of the dotted lines performed significantly below average. CI recipients presenting with etiologies on the right part of the dotted lines performed significantly better than average. Adapted from Blamey et al (in press).

The four factors, used in the four-factor general linear model of Blamey et al (in press) were: *duration of s/p HL*, defined as the time in years between the onset of s/p HL and the date of implantation; mean and median *durations of s/p HL* were 7.4 years and 3.2 years, respectively (ranges: 0–60 years, standard deviation: 9.8); mean *age at onset of s/p HL* was 50 years (standard deviation: 17.3); *duration of implant experience* was defined as the time elapsed between the date of first activation and the dates of testing. It ranged from 2 months to 12 years; fifteen *etiologies* were defined. They are detailed in Figure 2 *Age at implantation* ranged from 17 years to 93 years (mean: 58, standard deviation: 15.8). It was not included in the four-factor general linear model of Blamey et al (in press) because it had less effect than *age at onset of s/p HL*.

In addition to duration of s/p HL, age at onset of s/p HL, duration of CI experience, and etiology, several pre-, per-, and post-operative factors were added to the statistical analyses.

The preoperative factors were:

*gender*. There were 1017 females, 820 males, and 414 patients with missing gender data.
*education level*, corresponding to the age at which the subject stopped studying. This factor was partitioned into ranges: stopping before the age of 12 years, before the age of 18 years, or continuing after 18 years old. These three ranges encompassed 21 subjects, 501 subjects, and 518 subjects, respectively, plus 1211 patients with missing data.
*duration of moderate hearing loss (mHL)*, lasted from the onset of mHL to the onset of s/p HL. The ranges used for the analyses were: 0–4, 5–9, 10–14, 15–19, 20–24, 25–34, 35–44 and over 45 years. The mean duration of mHL was 17 years (range: 0 in case of sudden hearing loss to 74 years, standard deviation: 14.6 years).
*preoperative HA use*. Centres reported whether the subject was using HAs, bilaterally, or monaurally, at the time of implantation. Four groups were defined: patients not using any HA, patients with a HA on the implanted side, patients with a HA on the ear contralateral to the implanted side, and patients wearing two HAs. These four ranges encompassed 429 subjects, 289 subjects, 386 subjects and 712 subjects, respectively (the rest had missing data).
*pure tone average (PTA) of the implanted ear, and the better PTA of the two ears*. The latter will be called PTA of the better ear. PTAs represented the mean of unaided residual hearing levels in decibels (dB HL) at 500, 1000, 2000 Hz, for all centres. Four ranges were used: 40–49, 50–74, 75–99, and 100+dB HL.
*hearing loss (HL) at 500 Hz*. This variable was included to test whether residual low frequencies were more relevant to maintaining functional auditory pathways. Similarly to PTA, HL at 500 Hz was considered for both the implanted ear and for the better ear. The ranges used were the same as for PTA.
*preoperative speech scores in quiet*. These were aided speech scores before implantation. A percentile rank for each patient within each centre was calculated to allow for differences in test type (phonemes, monosyllabic words, dissyllabic words, sentences) in different languages and different levels of presentation (from 55 to 75 dB SPL). Using ranking removes differences in clinical practice without removing the relative differences between patients within each centre [1].
*date at implantation*. Modifications of coding strategies since 2002 were tested indirectly through the date at implantation. Three ranges were used: 2002–2004, 2005–2007, 2008–2011. These three ranges encompassed 345 subjects, 822 subjects, and 1083 subjects, respectively (one date was missing).
*implanted ear*. The implanted ear was classified as the better ear, the worse ear, or similar when both ears had the same amount of residual hearing. The better ear was implanted in 611 cases, the worse ear in 1142 cases, and the two ears had similar residual hearing in 294 cases (the rest had missing data).

Peroperatively, only one factor was studied:

*surgical approach*. Cochleostomy and round window approaches were compared. They were performed in 1119 cases and 425 cases, respectively (information was missing in 707 cases).

The postoperative factors were:

*CI brand*. CIs from four different manufacturers were represented in the dataset. Speech processors for Advanced Bionics were Auria and Harmony, for Cochlear processors included models from Esprit3G to CP810, for Med-el were Tempo+ and Opus 2, and for Neurelec the Digisonic SP processor.
*angle of insertion of the electrode array*: Depth of electrode array insertion was expressed as an angle divided into three ranges: <370°, 370–539°, and ≥540°. It ranged from 135° to 730°.
*percentage of active electrodes*. The number of active electrodes reported at the first testing was expressed relative to the total number of electrodes available on the electrode array, as the total number varies with the CI brand. The ranges used were: ≤70%, 71–85%, and >85%. The minimum percentage of active electrodes was 15%.

### Statistical analyses of speech scores in quiet

Postoperative speech scores in quiet were transformed into percentile ranks for each patient within each centre. Using ranking removes differences in clinical practice without removing the relative differences between patients within each clinic. Indeed, for each clinic, the distribution varied uniformly from 0 to 100. The best performers from each centre had a percentile rank close to 100, and the poorest performers from each group had a percentile rank close to 0. The ranked data of the centres were combined for the global analysis. Preoperative and postoperative scores were ranked separately. Ranked postoperative scores were used as the dependent variables of the statistical analyses described below.

Each new factor that we wanted to test was added into the four-factor unbalanced analysis of variance using the General Linear Model (GLM; Minitab version 12), previously described in Blamey et al (in press) to create fifteen five-factor ANOVAs. Briefly, a GLM studies the influence of various independent factors on a dependant variable. The four-factor ANOVA described in Blamey et al (in press) was based on main well-established general factors (the independent factors), known to influence CI speech performance (the dependant variable). These four common factors were duration of s/p HL, age at onset of s/p HL, duration of CI experience, and etiology. In the present study, we wished to explore the influence of 15 other factors that have been less studied. Because entering 19 different independent factors was not possible with the software used (Minitab version 12) and because interpretation of the results would have been complicated, we entered into the former four-factor GLM a single new factor once at a time, leading to 15 different five-factor GLMs. From these 15 analyses, only factors with p≤0.001 were selected. These significant factors were further included in a single GLM analysis (a sixteenth analysis) to investigate the interrelations between them and produce a new model of auditory performance.

### Statistical analyses of speech scores in noise

Postoperative speech scores in noise were ranked separately for each patient within each centre, and independently of scores in quiet. The noise used varied across centres from a cocktail party, to a pink noise, or a speech shaped noise, but was the same for all patients of the same center. Scores in noise at T1 and T2 were considered independent scores for the same patient, and used as dependent variables in the analysis used to explore the new model of auditory performance (the sixteenth analysis). This last analysis is detailed further in the Results section.

## Results

### Factors influencing CI outcome

The results from the 15 different five-factor GLM analyses are shown in Table 1. Only the results related to the new fifth factor are shown, the values for the four usual factors being stable across the 15 five-factor GLM analyses. The F ratio was used to test the significance of the fifth factor in each analysis. The number of degrees of freedom for the numerator is one less than the number of ranges for the factor. The number of degrees of freedom for the error term (dfe) differs from one analysis to another because some data were missing for some factors. The value of (dfe+32+df+1) is equal to the number of data points in each analysis (32 is the number of degrees of freedom used by the other factors in the analysis). Because of the large number of data points in the analyses, we considered p<0.001 as statistically significant. Factors with 0.05>p>0.001 are referred to as “marginally significant” in the following discussion to provide the reader with some insight into the weaker trends in the data. Inclusion of the marginally significant factors in the new model of auditory performance would have complicated the model for a relatively small increase in its predictive power.

**Table 1:** Results from the 15 five-factor GLM analyses.

Factor tested	F(df, dfe)	Significance p
Gender	(1, 2533) = 0.97	0.325
Education level	(2,1685) = 1.40	0.246
Duration of moderate HL	(7, 2155) = 7.44	0.000*
Hearing aid use	(3, 2833) = 6.99	0.000*
PTA of the implanted ear	(3, 2979) = 4.08	0.007
PTA of the better ear	(3, 3000) = 8.46	0.000*
HL at 500 Hz of the implanted ear	(3, 2860) = 3.98	0.008
HL at 500 Hz of the better ear	(3, 2881) = 7.43	0.000*
Ranked preoperative scores	(4, 2897) = 17.06	0.000*
Date at implantation	(2,3135) = 5.20	0.006
Implanted ear: better ear, worse ear	(2,2984) = 2.63	0.072
Surgical approach	(1, 2380) = 4.18	0.041
Brand	(3,2995) = 41.19	0.000*
Angle of insertion of the electrode array	(2,469) = 3.93	0.020
Percentage of active electrodes	(2,2273) = 35.77	0.000*

Gender, the implanted ear (worse, better, similar), and education level had no significant effect (p>0.05) and were not included in later analyses. The effects of PTA and HL at 500 Hz of the better ear produced p values lower than those of the implanted ear. We selected PTA in the better ear for the second analysis ahead of HL at 500 Hz, because the F factor was bigger (8.46 vs. 7.43). The effect of preoperative score was significant, but as demonstrated in Table 2, it was significantly influenced by other factors (age at onset of s/p HL, PTA of the better ear, HA use, p<0.001), and was therefore not included as an independent variable in the sixteenth analysis used to test the new model of auditory performance. Indeed, a fundamental assumption to perform a GLM analysis requires that the factors entered are independent. Date at implantation, the surgical approach, and the angle of insertion had marginally significant effects (0.05>p>0.001). The other new factors that had a significant influence (p<0.001) on CI outcomes were duration of mHL, HA use, CI brand, and percentage of active electrodes. Only these new relevant factors were consequently entered in the sixteenth analysis aiming to test the new model of performance over time (Figure 1).

**Table 2:** Results of a GLM analysis using ranked preoperative speech scores as dependent variable, and main preoperative factors (determined from Table 1).

Factor	Degree of freedom	Sum of squares	Mean squares	F	p
Age at onset of s/p HL	6	11221.1	2661.6	5.21	0.000
Duration of s/p HL	7	3112.3	416.2	0.81	0.575
Etiology	14	19822.6	1049.0	2.05	0.012
Duration of mHL	7	4001.1	571.6	1.12	0.349
PTA of the better ear	3	131808.3	30122.6	58.94	0.000
HA use	3	42184.3	13667.8	26.75	0.000
Error	1036	529436.7	511.0		
Total	1076	741586.5			

A further multivariate GLM analysis (the sixteenth analysis), including all of the significant factors (9 in total), was conducted to determine the relative influence of these factors in the new model of performance over time shown graphically in Figure 1. Durations of s/p HL and mHL were treated as continuous covariates in the GLM analysis instead of categorical variables as they were in the previous analyses, to measure the rates of decrease (slopes) of auditory performance over time. Duration of s/p HL was nested within HA use to test the hypothesis that the negative effect of s/p HL might be affected by hearing aid use. The results of this analysis are provided in Table 3. All the factors studied still had a significant effect in the new analysis, except for the use of HA on its own and PTA of the better ear (marginally significant, and therefore not included in the final version of the model).

**Table 3:** Results of a GLM analysis testing the new model of auditory performance for speech in quiet.

Factor	Degree of freedom	Sum of squares	Mean squares	F	p
Age at onset of s/p HL	6	32144.8	5838.9	8.59	0.000
Etiology	14	14278.5	1382.3	2.03	0.013
Duration of CI experience	5	155534.2	33874.9	49.81	0.000
PTA of the better ear	3	12383.0	2804.4	4.12	0.006
HA use	3	8947.9	1196.2	1.76	0.153
Brand	3	53601.5	11313.2	16.63	0.000
Percentage of active electrodes	2	23669.7	12169.8	17.89	0.000
Duration of s/p HL(HA use)	4	40819.3	11120.1	16.35	0.000
Duration of mHL	1	17231.6	17231.6	25.34	0.000
Error	1894	1288090.5	680.1		
Total	1935	1646701.0			

Durations of severe to profound hearing loss and of moderate hearing loss were analysed as continuous (regression) variables. A separate regression coefficient was calculated for each hearing aid use category.

### Effects of age at onset of s/p HL, etiology, duration of CI experience

The new analysis did not modify the relative importance of each of the main factors already studied in Blamey et al (in press), i.e. using a four-factor GLM analysis with categorical variables in Blamey et al (in press), or a nine-factor GLM analysis with two continuous variables (durations of s/p HL and of mHL) provided equivalent results for the F and p values of age at onset of s/p HL, etiology, and duration of CI experience. These values were F = 35.31, p<0.0001 for CI experience in Blamey et al versus F = 49.81, p<0.0001 in the new analysis. F = 17.91, p<0.0001 for age at onset of s/p HL in Blamey et al versus F = 8.59, p<0.0001 in the new analysis. F = 2.46, p = 0.002 for etiology in Blamey et al versus F = 2.03, p = 0.013 in the new analysis. Note that duration of s/p HL was treated as a continuous regression variable in the new analysis and as a categorical factor in the original analysis, so the F values for this factor were not comparable on theoretical grounds.

### Effect of duration of s/p HL, influenced by the use of HAs

When nested with duration of s/p HL, each group of HA use had a significant effect. The regression analysis showed that wearing no HA before implantation induced a loss of CI speech performance of 0.83% per year of s/p HL (p<0.001), using one HA on the future implanted ear induced a loss of CI speech performance of 0.64% per year of s/p HL (p = 0.002), using one HA on the ear contralateral to the implanted ear induced a loss of CI speech performance of 0.49% per year of s/p HL (p = 0.017), and using two HAs induced a loss of CI speech performance of 0.45% per year of s/p HL (p = 0.003). The slopes used in Figure 1 are the actual values derived from the nested GLM regression analysis.

### Effect of duration of mHL

The GLM regression analysis for duration of mHL showed that CI speech performance reduced by 0.23% per year of mHL (p<0.001). The slope used in Figure 1 is the actual value derived from the GLM regression.

### Effect of PTA of the better ear

**Figure 3:**
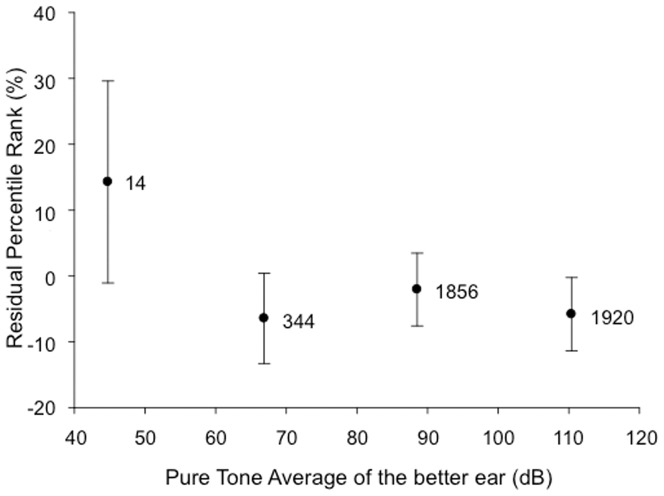
Significant effect of Pure Tone Average thresholds of the better ear on the residual percentile rank. Error bars indicate +/− two standard errors of the mean for each pure tone average range (approximately equivalent to the 95% confidence interval for each mean value shown on the graph; if two mean values fall within one error bar, then the means are not significantly different (p>0.05)). The numbers next to each symbol indicate the number of data points in that range.

The effect of PTA of the better ear was marginal (Table 3, F = 4.12, p = 0.006). The results for the ranges used are represented in Figure 3. Patients with residual hearing better than 50 dB HL in the better ear had better CI speech scores. However, the variance of this group is large due to the small number of patients and possibly to the inclusion of patients presenting with auditory neuropathy spectrum disorder, who performed below the average outcome (Blamey et al, in press and Figure 2. Patients with severe HL and profound HL displayed similar CI performance.

### Effect of CI brand

**Figure 4:**
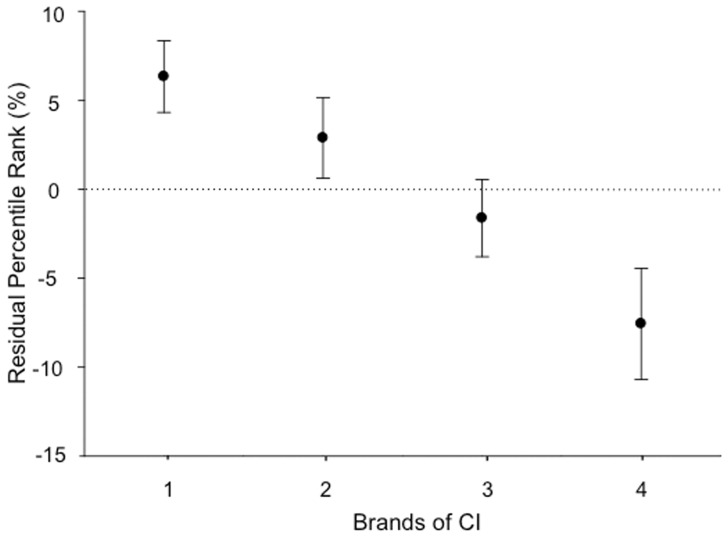
Significant effect of brands of CI on the residual percentile rank. Error bars indicate +/− two standard errors of the mean for each CI brand (approximately equivalent to the 95% confidence interval for each mean value shown on the graph; if two mean values fall within one error bar, then the means are not significantly different (p>0.05)). The numbers of data points for each brand were not indicated to avoid potential identification of the individual brands.

The effect of CI brand was significant (Table 3, F = 16.63, p<0.001). The results are represented in Figure 4. The horizontal dotted line represents the average performance (50% of speech performance for the ranked scores in quiet). Although the difference between the mean percentile rankings of the highest and 2 lowest brands was significant, the mean scores of the highest and the lowest brand differed by only 14%.

### Effect of percentage of active electrodes

**Figure 5:**
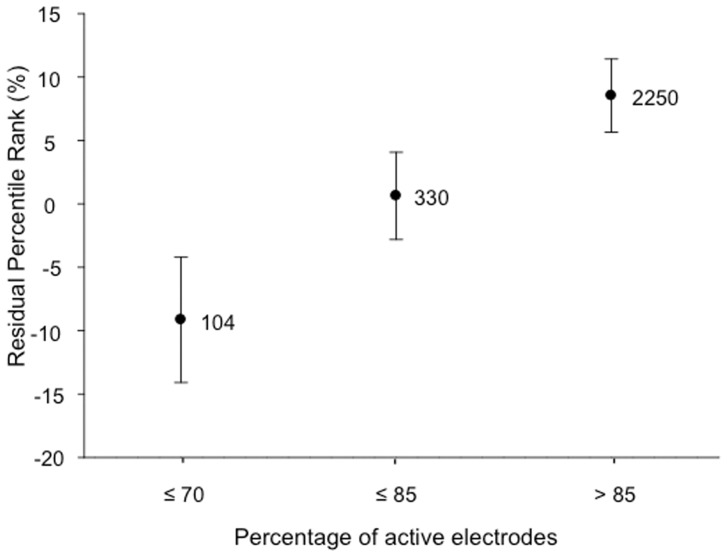
Significant effect of percentage of active electrodes on the residual percentile rank. Error bars indicate +/− two standard errors of the mean for each range (approximately equivalent to the 95% confidence interval for each mean value shown on the graph; if two mean values fall within one error bar, then the means are not significantly different (p>0.05)). The numbers next to each symbol indicate the number of data points in that range.

The effect of percentage of active electrodes was significant (Table 3, F = 17.89, p<0.001). Figure 5 shows the residual CI speech performance versus the percentage of active electrodes. A rise in performance was observed when the overall number of electrodes increased.

### Defining a new model of auditory performance in quiet over time

The multivariate GLM analysis enabled a determination of the relative influence of the nine most relevant factors in a new model of performance. This model, proposed in Figure 1 for a hypothetical “average patient”, starts at the beginning of mHL. It is important to note that “auditory performance” on the vertical axis of Figure 1 refers to the expected percentile rank with a CI if the hypothetical patient were to receive a CI. Mean expected auditory performance ranking with a CI for very short periods of mHL and s/p HL and no CI experience is about 65% relative to the mean ranking for all patients, which is always 50%. The percentages of performance lost per year of mHL or per year of s/p HL were derived from the regression analyses for these variables included in the GLM analysis. The slope of the relevant regression line has units of percentile rank change per year: during the period of mHL, mean expected CI auditory performance ranking slowly decreases by 0.23% per year. We have assumed about 20 years of mHL for the hypothetical patient in Figure 1, so auditory performance decreases down to 60%, where the hypothetical patient presents with a s/p HL. During the period of s/p HL, the decrease in mean expected ranking depends on HA use, at about 0.45% per year if 2 HAs are worn, and 0.89% if no HAs are worn. We have assumed about 10 years of s/p HL in Figure 1. The final stage represents the post-operative learning curve related to CI experience. The expected outcome for an individual patient will be influenced by the use or absence of HAs, the amount of residual hearing, the age of occurrence of the various events, the actual duration of mHL and s/p HL, the brand of CI, and the percentage of active electrodes postoperatively. Although explicit experimental measures, such as fMRI, are not routinely available, the effects of peripheral and central reorganization are partially taken into consideration by the factors tested. In total, the new model accounts for 22% of the variance in the data. The former model (four-factor GLM analysis) accounted for 10% of the variance in the present data set (Blamey et al in press).

### Testing the new model of auditory performance in noise over time

The ranked speech perception scores in noise were subjected to the GLM analysis developed to test the new model of auditory performance to determine whether the nine most relevant factors had similar effects in quiet and in noise. After allowing for missing data, there were 1037 data points in the analysis of performance in noise. The results of this analysis are shown in Table 4. Duration of CI experience still had the greatest effect. The effect of CI brand in noise was this time marginally significant and much smaller than in quiet (F(3,995) = 3.80, p = 0.01 and F(3,1894) = 16.63, p<0.001, respectively). The percentage of active electrodes had no significant effect in noise. The relative importance of duration of mHL was greater in noise, with a 50% steeper slope of decrease of auditory performance per year compared to the slope for auditory performance in quiet (−0.32% versus −0.23%). The slopes related to duration of s/p HL, ranging from −0.85% to −0.49%, were similar to those observed for auditory performance in quiet. In total, the percentage of variance accounted for by the new model in noise was the same as in quiet (22%).

**Table 4:** Results of the new GLM analysis using ranked speech scores in noise with a CI as the dependent variable.

Factor	Degree of freedom	Sum of squares	Mean squares	F	p
Age at onset of s/p HL	6	27576.8	4499.3	6.56	0.000
Etiology	14	9395.5	697.0	1.02	0.434
Duration of CI experience	5	88794.2	18792.7	27.41	0.000
PTA of the better ear	3	8680.0	2893.3	4.22	0.006
HA use	3	6826.4	371.0	0.54	0.654
Brand	3	8887.2	2604.9	3.80	0.010
Percentage of active electrodes	2	1563.7	1838.3	2.68	0.069
Duration of s/p HL(HA use)	4	21207.3	5150.8	7.51	0.000
Duration of mHL	1	20823.8	16580.9	24.18	0.000
Error	995	682242.0	685.7		
Total	1036	875997.0			

## Discussion

By including a large number of patients (2251) from 15 different centres, and removing the differences that may exist between clinical practices (speech material, level of presentation used) by using percentile ranking, the present study investigated the effects of variables that are routinely accessible from clinical files, to find those that have an effect on CI outcome.

### Factors with little or no influence on CI outcome

Gender and level of education did not influence CI speech performance of postlinguistically deaf adults. The inclusion criteria of the present study paid particular attention to distinguish level of education and language acquisition. The age at beginning of s/p HL had to be later than 15 years old, in order to avoid bias related to potentially delayed development of speech processing in severely deaf children [23].

Implanting the better ear [24] or the worse ear [25], defined on PTA criteria only, had no effect [26]. In the sample studied, the clinical practice that was most frequently used was to implant the worse ear (56% of cases). The better ear was implanted in 30% of cases, and the 14% remaining concerned ears with symmetric PTA. It is likely that the actual choice of the side to implant was based on patients' reports rather than PTA. The ear with the better PTA is not always the ear with the more usable acoustic hearing, and the ear with the shortest duration of deafness is often preferred when aided performances are similar [27]. Preserving efficient residual hearing of the better ear (i.e. implanting the worse ear) is nowadays a general consensus, enabling the use of a HA contralateral to the CI [11], [28], [29]. The present study confirms that implantation of the poorer ear is unlikely to reduce the CI outcome significantly. The level of residual hearing of the better ear had a significant influence on CI outcome (Table 2), as discussed latter.

Surgical approach is a highly debated topic. Various publications defend one technique over the other one [15]–[18], [30]. In this study, surgical approach had a marginally significant effect on outcomes (p = 0.041). Cochleostomy was practiced in 73% of cases, and the round window approach in 27%. Except for some electrode arrays for which a specified approach is recommended [17], the best approach may be the one that the surgeon controls best, depending on his/her surgical practice and habits, and on the local anatomy of the middle ear.

There were 504 data points from 4 different cochlear implant centers in the analysis for angle of insertion of the electrode array in Table 1. So far, the literature has been consistent in indicating the importance of studying electrode array placement, on postoperative CT scans, for CI outcome [19], [20], [31], [32]. Predictors of good performance are: a greater number of electrodes within the scala tympani, an absence of translocation from the scala tympani to the scala vestibuli, a not excessively deep insertion, and a reduced distance to the modiolus. However, the present study found only a marginally significant effect of the angle of insertion (p = 0.02). It is noted that visibility of the electrode array and electrode contact positions may vary between scanners [33], that the angle of insertion may not correspond directly to the electrode array placement [32], and that the evaluation of the angle of insertion may require specific training of the radiologists involved.

The date at implantation was marginally significant (p = 0.006). However, Blamey et al (in press) and Zeng et al [34] suggest that the greater improvements in performance over time, compared with older studies [1], were related in part to improvements in coding strategies. Most of the major steps were taken before 2002, the beginning of the inclusions in the present study, and corresponded to a switch from F0F2, F0F1F2 and MPEAK strategies to Continuous Interleaved Sampling (CIS), and spectral-maxima (ACE or N of M) strategies [34], [35]. Between 2002 and 2011, the coding strategies have remained much the same although other new sound processing features have been introduced, such as ADRO (Adaptive Dynamic Range Optimization) [8], and noise reduction algorithms [36], [37], whose benefits are not explored by speech tests in quiet at conversational levels.

### New factors with strong influence on CI outcome

Effects of age at onset of s/p HL, etiology, and duration of CI experience were similar to those found in Blamey et al (in press). They accounted for 10% of the variance in this previous study (four-factor GLM analysis) (Blamey et al in press).

Audiometric features, PTA of the implanted ear, PTA of the better ear, residual hearing at 500 Hz of the implanted ear, and residual hearing at 500 Hz of the better ear, had consistent significant effects (Table 1). The audiometric feature which had the bigger F value was PTA of the better ear (F(3, 3000) = 8.46, p<0.001), compared with residual hearing at 500 Hz of the better ear (F(3, 2881) = 7.43, p<0.001). These results suggest that using PTA, averaging the audiometric thresholds at 500, 1000 Hz and 2000 Hz, to define the severity of the hearing loss and the efficiency of the residual hearing, is a valuable practice, as audiometric thresholds at the low frequency studied (500 Hz) had a slightly smaller effect on the overall CI performances. The F values for the implanted ear, PTA and threshold at 500 Hz, were about half the corresponding values for the better ear. These results may indicate that speech performance with a CI does not rely more on the peripheral structures of the implanted ear, but more on the integrity of central processing. This possibility is consistent with the strong relationship between PTA in the better ear and pre-operative speech perception scores (Table 2). Whichever ear is implanted, what seems to matter is that the brain was not deprived of auditory inputs pre-operatively [26], [38]. The redundant ascendant crossed auditory pathways seem to enable auditory processing independently of the side of the electric stimulation and of the asymmetric speech processing of the brain (see [39] and [40] for reviews of asymmetric speech processing). However, as shown in Figure 3, mean CI performances were similar for groups of recipients with unaided PTA of more than 65 dB. This result must be considered in the context of the other variables in the analysis that can modify the observed effects of the degree of residual hearing. For example, auditory processing and central preservation probably depend more on aided thresholds than unaided PTA, thus HA use may tend to reduce the observed effect of PTA. Durations of s/p HL and mHL are also included in the analysis as covariate factors which account for the effects of degree of hearing loss to a large extent. Thus inclusion of these interrelated variables in a single multivariate analysis may have acted to reduce the significance of the PTA factor in Table 3 relative to Table 1 where there were fewer interrelated variables.

Hearing aid use had a strong effect through its influence on the slope of the duration of s/p HL regression (Table 3 and Figure 1). As hypothesized, not using any HA accelerated the central and peripheral modifications induced by auditory deprivation. The amount of reduction of CI speech performance was 0.83% per year for the patients who used no HA during the period of s/p HL, while it was 0.45% per year for the patients who used two HAs during the period of s/p HL. These results confirm that inputs from HAs may slow down the pathological reorganization of auditory pathways induced by hearing loss [41], [42]. Using only one HA on the future implanted ear was linked to a marginally greater reduction of CI speech performance than using only one HA on the ear contralateral to the implanted ear (0.64% per year of s/p HL vs 0.49%). Because general practice is to preserve the ear with the more efficient residual hearing [11], [28], [29], we may hypothesize that if the ear chosen to be implanted was the only one using a HA, the contralateral ear was profoundly deaf and probably presented with a much longer duration of s/p HL [27]. The central and peripheral auditory wiring might have been poorer in this case than when the HA was worn on the non-implanted ear. This may explain the greater negative effect of using one HA on the future implanted ear.

CI brand had a significant effect in the model of Figure 1 (F(3, 1894) = 16.63, p<0.001). It should be noted that these results are an average picture of the situation between 2002 and 2011. Some brands have already introduced new processors that were not included in this study. Technical improvements are continuing, and current performance may be different from this average over the last ten years. There was 14% difference between the best and poorest device in this analysis. This result may be considered rather small compared to the 0–100 range that exists in CI speech performance in quiet. An improvement to a particular device may easily change its positioning in the next decade. It is also important to note that other studies have found different results when comparing brands of CI [43]. The performance of each brand may vary depending on the characteristic tested (e.g. dynamic range, noise reduction strategy), the speech material used [43], and the test conditions as suggested by the present analysis of speech perception in noise. Another factor affecting these results may be the strategy used. In the present database, this item was not recorded, supposing that the default strategy of every brand was used in most of the cases. Other elements apart from performance, such as reliability, design of the electrode array and placement in the cochlea, may also be taken into consideration to evaluate a CI.

The percentage of active electrodes had a strong effect (F(2, 1894) = 17.89, p<0.001). Having more than 85% of active electrodes conferred a significant advantage in speech perception. A smaller percentage could reflect the number of electrodes inserted, and/or of deactivated electrodes (high impedance, facial stimulation, uncomfortable sensations) [22]. Reducing the percentage of active electrodes means that the actual number of intra-cochlear sites available for stimulation is reduced, indirectly reflecting the neural population stimulated [21]. It is important to note that the absolute number of electrodes was not studied here, because it is confounded with the other factors that differentiate the CI devices from different companies.

The negative effect of duration of mHL on auditory processing [2] was confirmed (F(1, 1894) = 25.34, p<0.001). The reduction of CI speech performance per year of mHL (0.23%) was smaller than the reduction during duration of s/p HL. Using HAs during the period of mHL may also slow this reduction (this information was not accessible in the present database). In Lazard et al [2], it was shown that non-speech sound processing (i.e. environmental sound processing) decreased with duration of mHL, releasing cognitive resources recycled to process phonology. A decline in phonological processing was also observed, but it was correlated with duration of s/p HL. It was proposed that cerebral plasticity prioritized reorganization in favor of oral communication. The delayed decline of phonological processing was related to a sustained reinforcement by lipreading.

### Using the new model for speech perception in noise

The usual factors (duration of CI experience, age at onset of s/p HL) had the same importance in noise and in quiet. However, the results of the analysis in noise may be biased by the selection of only the best patients for testing in noisy conditions. Usually, poor performers are less likely to be tested in noise than the better performers, to avoid them having to face listening conditions that are too difficult. There were only about half as many data points in Table 4 as in Table 3, as indicated by the total degrees of freedom. The reduced significance of the percentage of active electrodes may come from this biased selection. It seems unlikely that in noise patients with a small number of active electrodes perform on average as well as patients with a greater number of active electrodes, although it is also possible that the noise obliterates some of the fine spectral detail that can be used with a larger number of electrodes. The modification of the importance of brand may also be explained by the selection of the best performers, or it may be that the differences across brands are dampened in difficult listening conditions, showing that speech understanding in noise with a CI remains challenging whatever the device. The increased effect of duration of mHL in noisy conditions may be related to cerebral reorganization of non-speech sound and environmental sound processing. Indeed, the cortical activation of some areas usually involved in non-speech sound and environmental sound processing starts to decrease during the period of mHL [2], [41]. This last result may emphasize the importance of promoting the use of HAs to maintain the functional processing of both speech and environmental sounds in a noisy world.

## Conclusions

Several questions were addressed in the present study. In particular, the model used did not find any significant effect of gender, level of education, or ear of implantation based on worse or better PTA. Surgical approach was marginally significant.

An older model including only 4 factors (duration of s/p HL, age at onset of s/p HL, etiology, and duration of CI experience) accounted for 10% of the variance in the same dataset as used in the present study (Blamey et al, in press). The new model described in the present study, and including the nine most significant factors among 15, accounted for 22% of the variance, and shed light on the roles of mHL, s/p HL and HA use. A part of the unexplained 78% of the variance is likely to be due to test/retest reliability of the speech perception measures used, some of which have only a relatively small number of test items. Indeed, the fewer items in a test, the greater the variability. The rest of the variance remains unexplained, but high order cognitive reorganization may be involved [42], [44], [45], as well as other variables not accessible from clinical information routinely collected.
